# Dexamethasone-Induced Myeloid-Derived Suppressor Cells Prolong Allo Cardiac Graft Survival through iNOS- and Glucocorticoid Receptor-Dependent Mechanism

**DOI:** 10.3389/fimmu.2018.00282

**Published:** 2018-02-15

**Authors:** Yang Zhao, Xiao-Fei Shen, Ke Cao, Jie Ding, Xing Kang, Wen-xian Guan, Yi-tao Ding, Bao-rui Liu, Jun-Feng Du

**Affiliations:** ^1^The Comprehensive Cancer Center of Drum Tower Hospital, Medical School of Nanjing University, Clinical Cancer Institute of Nanjing University, Nanjing, China; ^2^Department of General Surgery, Nanjing Drum Tower Hospital, The Affiliated Hospital of Nanjing University Medical School, Nanjing, China; ^3^Department of Critical Care Medicine, Nanjing Drum Tower Hospital, The Affiliated Hospital of Nanjing University Medical School, Nanjing, China; ^4^Department of General Surgery, PLA Army General Hospital, Beijing, China

**Keywords:** myeloid-derived suppressor cells, immune tolerance, dexamethasone, iNOS, glucocorticoid receptor

## Abstract

How to induce immune tolerance without long-term need for immunosuppressive drugs has always been a central problem in solid organ transplantation. Modulating immunoregulatory cells represents a potential target to resolve this problem. Myeloid-derived suppressor cells (MDSCs) are novel key immunoregulatory cells in the context of tumor development or transplantation, and can be generated *in vitro*. However, none of current systems for *in vitro* differentiation of MDSCs have successfully achieved long-term immune tolerance. Herein, we combined dexamethasone (Dex), which is a classic immune regulatory drug in the clinic, with common MDSCs inducing cytokine granulocyte macrophage colony stimulating factor (GM-CSF) to generate MDSCs *in vitro*. Addition of Dex into GM-CSF system specifically increased the number of CD11b^+^ Gr-1^int/low^ MDSCs with an enhanced immunosuppressive function *in vitro*. Adoptive transfer of these MDSCs significantly prolonged heart allograft survival and also favored the expansion of regulatory T cells *in vivo*. Mechanistic studies showed that inducible nitric oxide sythase (iNOS) signaling was required for MDSCs in the control of T-cell response and glucocorticoid receptor (GR) signaling played a critical role in the recruitment of transferred MDSCs into allograft through upregulating CXCR2 expression on MDSCs. Blockade of GR signaling with its specific inhibitor or genetic deletion of iNOS reversed the protective effect of Dex-induced MDSCs on allograft rejection. Together, our results indicated that co-application of Dex and GM-CSF may be a new and important strategy for the induction of potent MDSCs to achieve immune tolerance in organ transplantation.

## Introduction

The ultimate goal for organ transplantation is to achieve long-term acceptance without the need for immunosuppressive drugs (1). Cellular immunotherapy is a promising alternative for preventing allograft rejection and adoptive transfer of immune cells has been tried to establish immune tolerance in animal models (2–4). Our previous results showed that adoptive transfer of adaptive immune cells, regulatory T cells (Treg), can help to establish mixed bone marrow chimeras and lead to the prolongation of intestinal allograft survival (5). However, the possibility of adoptive transfer of other immune cells such as innate immune cells to achieve immune tolerance has not been fully elucidated.

Myeloid-derived suppressor cells (MDSCs) are a heterogeneous population of innate immune cells generated from immature myeloid cells in the bone marrow and were first characterized in tumor-bearing mice or in patients with cancer (6). With the increased understanding of MDSC development, it is now clear that pathological situations such as inflammation and tumor development can lead to a partial block in the differentiation of immature myeloid cells into mature myeloid cells, resulting in the expansion of MDSCs (6). In mice, the phenotype of MDSCs was CD11b (also known as integrin-αM) and granulocyte differentiation antigen-1 (Gr-1), and in humans, their phenotype is Lin^−^HLA^−^DR^−^CD33^+^ or CD11b^+^CD14^−^CD33^+^ (6). Once generated in a pathological context, MDSCs were able to control T-cell immune response through the upregulated expression of immune suppressive factors such as arginase, inducible nitric oxide synthase (iNOS), nitric oxide, and reactive oxygen species. Thus, despite early evidence of MDSCs in the control of tumor development, accumulating results have also suggested a critical role of MDSCs in the amelioration of allogenic transplantation based on their immune suppressive function (4, 7). Manipulating both the development and function of MDSCs has been suggested to be a potential therapeutic target to achieve immune tolerance (8). However, although numerous studies have identified multiple signaling pathways to contribute to the induction and function of MDSCs *in vivo* and *in vitro*, none of them have been applied to clinical trials possibly due to the instability of MDSCs *in vivo*.

Myeloid-derived suppressor cells can be induced from bone marrow progenitors *in vitro*, and the most common way to induce MDSCs *in vitro* is the use of granulocyte macrophage colony stimulating factor (GM-CSF) at high concentrations for 4 days (9). In addition, *in vitro*-generated MDSCs by GM-CSF have also been shown to prolong the survival of pancreatic islet allograft upon adoptive transfer (7), which provides promising prospects of cellular immunotherapy based on *in vitro*-generated MDSCs in the context of transplantation. Dexamethasone (Dex) belongs to the family of glucocorticoid and has been widely used in treating inflammatory disorders (10). Dex can not only inhibit the production of inflammatory cytokines *in vivo* but also modulate the phenotype and/or function of both classic innate and adaptive immune cells such as dendritic cells (11), macrophages (12), and Treg (13). However, the role of Dex in the modulation of MDSCs has not been fully addressed. In the present study, we sought to determine whether the use of Dex together with GM-CSF can promote the differentiation of MDSCs *in vitro* with an enhanced immunosuppressive ability, and also explore whether adoptive transfer of these *in vitro*-induced MDSC can help to achieve immune tolerance *in vivo*.

## Materials and Methods

### Mice

BALB/c, C57BL/6 (B6), and CD45.1^+^ B6 mice were purchased from Model Animal Research Center of Nanjing University (Nanjing, China). All the mice were bred and maintained in the condition of specific pathogen-free. Male 8- to 10-week-old littermate mice were used for experiments. iNOS knockout (KO) mice (B6 background, CD45.2) were purchased from Jackson Lab. All the animal experiments were carried out following the Guidelines for the Care and Use of Laboratory Animals of Nanjing University and were approved by the ethical review committee of Nanjing University.

### Cell Culture

Bone marrow cells were acquired from tibias and femurs of B6 mice (CD45.1 or CD45.2) as described previously (14). Tibias and femurs were removed using sterile techniques, and bone marrow was flushed with PBS. Red blood cells were lysed with ammonium chloride. Total bone marrow cells were planted into 100-mm dishes (Corning, USA) for 2 h. The non-adherent cells were collected and were further cultured in the presence of 50 ng/ml GM-CSF, with or without Dex at different concentration as indicated in 60-mm-diameter dish (Corning, USA) in 4 ml of complete RPMI 1640 medium (containing 10% heat-inactivated FBS, 2mM L-glutamine, 10 mM HEPES, 20 µM 2-ME, 150 U/ml streptomycin and 200 U/ml penicillin) at 37°C, 5% CO_2_ for 4 days.

### T Cell Proliferation Assay

Splenic cells were labeled with 2 µM CFSE for 5 min in PBS at 37°C and were then washed with RPMI 1640. The labeled cells were further cultured with ConA (2 µg/ml) (15) in the presence of different doses of MDSCs as indicated for 72 h. Cell proliferation was assessed by flow cytometry after stained with anti-CD4 or anti-CD8 mAb.

### Flow Cytometry Antibodies and Reagents

Anti-mCD11b-FITC, anti-mCD11b-PE, anti-mGr-1-PE-Cy5, anti-mCD31-PE, anti-mF4/80-PE, anti-mCD11c-PE, anti-mI-Ab-PE, anti-mCD115-PE, anti-mCD274-PE, anti-mCD80-PE, anti-mCD86-FITC, anti-mCD115-PE, anti-mCD62L-PE, anti-mIgG-PE, anti-mIgG-FITC, and anti-mFoxp3-PE were purchased from BD Biosciences Pharmingen (San Diego, CA, USA). Anti-mCD8-PE-Cy5, anti-mCD4-FITC, and anti-mCD4-PE were purchased from eBioscience (San Diego, CA, USA). Anti-mCD45.2-PE-Cy5, anti-mCD45.1-APC, anti-mLy6G-PE, and anti-mCD11b-FITC were purchased from biolegend. Recombinant mouse GM-CSF was purchased from PeproTech (Rock Hill, NJ, USA). Dex, RU486 (16), bacterial lipopolysaccharide (LPS; *E. coli* 055:B5), and NG-monomethyl-l-arginine (L-NMMA) (17) were purchased from Sigma-Aldrich. Anti-CXCR2 mAb (16) and IgG isotype were purchased from R&D Systems (Minneapolis, MN, USA).

### NO Production Assay

The Griess reaction was used to measure nitrite production. Equal volumes of culture supernatants (100 µl) were incubated in 96-well plate with Griess reagent at room temperature for 10 min, and absorbance was determined at 550 nm in a microplate reader (Bio-Rad). Nitrite concentrations were calculated by comparing the absorbance values for the test samples to a standard curve generated by a serial dilution of sodium nitrite.

### Cardiac Allograft Transplantation

Hearts from Balb/c donors were transplanted heterotopically into the abdominal cavity of B6 recipients. Anastomoses were performed between the donor ascending aorta/pulmonary arties and the recipient descending aorta/inferior vena cava. The function of the cardiac graft was monitored daily by palpation through the abdominal wall. Graft loss was defined as no palpable beat. In some experiments, grafts were excised, fixed in 10% buffered formaldehyde, paraffin embedded, sectioned (3 µm), and stained with hematoxylin and eosin (H&E) as we described previously (5).

### Cell Isolation, Flow Cytometry, and Adoptive Transfer

Splenic cells and peripheral white blood cells were prepared as we described previously (5). For isolation of infiltrated cells in cardiac allografts, allografts were cut into small pieces and then digested for 40 min at 37°C with 400 U/ml collagenase IV (Sigma-Aldrich), 10 mM HEPES, and 0.01% DNase I (MP Biomedicals) in HBSS. Digested suspensions were passed through a nylon mesh. Then cells were collected after centrifugation and re-suspended in FACS staining buffer for cell surface marker staining. Samples were analyzed on a Beckman Coulter Epics XL benchtop FCM (Beckman Coulter) with FCS express software (*De Novo* Software, Thornhill, ON, Canada). CD11b^+^ Gr-1^int/low^ cells were sorted after *in vitro* culture using a FACSAria II (BD Biosciences) and were adoptively transferred into B6 recipient immediately after cardiac allograft transplantation (3 × 10^6^ per mouse, i.v.).

### Quantitative Real-time PCR

Total RNA of the indicated cells was extracted using RNAeasy mini kit (Qiagen) and reverse transcribed with Superscript II (Qiagen) according to the manufacturer’s instructions. The cDNA served as template for the amplification of target genes and the housekeeping gene (*HPRT*) by real-time PCR. Target gene expression was calculated using the comparative method for relative quantification upon normalization to *HPRT* gene expression. Primer sequences:

*HPRT* Forward: AGTACAGCCCCAAAATGGTTAAG,
  Reverse: CTTAGGCTTTGTATTTGGCTTTTC;
*iNOS* Forward: CACCAAGCTGAACTTGAGCG,
  Reverse: CGTGGCTTTGGGCTCCTC;
*GRα* Forward: ACTGCCCCAAGTGAAAACAGA,
  Reverse: GCCCAGTTTCTCCTGCTTAATTAC;
CXCR2 Forward: TCACAAACAGCGTCGTAGA,
  Reverse: GACAGCATCTGGCAGAATAG.

### Statistical Analysis

Data were reported as Mean ± SEM. Student’s *t*-test was used to compare the differences between two groups, and one-way or two-way ANOVA analysis was used for comparison among multiple groups. Survival studies were analyzed with the log-rank test. P < 0.05 was considered statistically significant.

## Results

### Dex Enhances the Differentiation and Suppressive Capacity of MDSCs Induced by GM-CSF *In Vitro*

**Figure 1:**
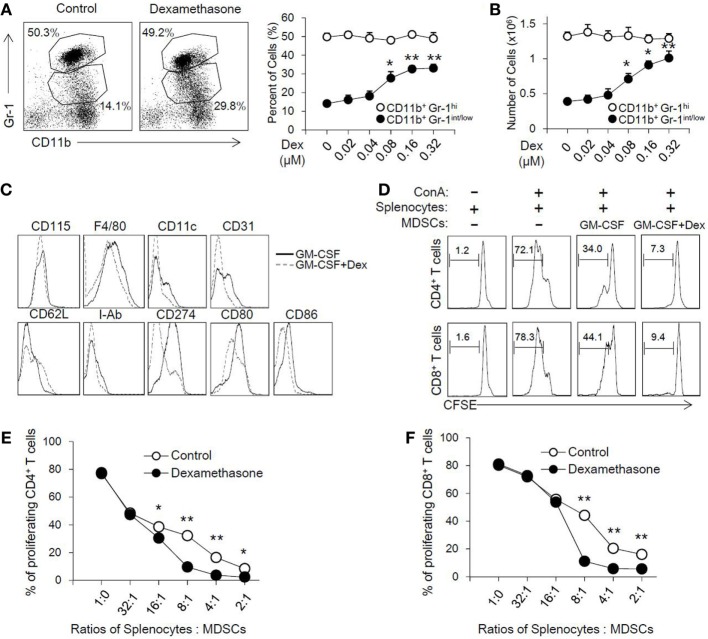
Dexamethasone (Dex) increases the cell number of CD11b^+^ Gr-1^int/low^ Myeloid-derived suppressor cells (MDSCs) with an enhanced suppressive function *in vitro*. MDSCs were induced from bone marrow cells in the presence of granulocyte macrophage colony stimulating factor (GM-CSF), with or without Dex for 4 days. The percentage **(A)** and cell number **(B)** of MDSCs induced by GM-CSF or GM-CSF + Dex were shown. The phenotype markers **(C)** of MDSCs induced *in vitro* were also summarized by flow cytometry. **(D)** CFSE-labeled splenic cells (2 × 10^5^ per well) were stimulated by ConA (2 µg/ml), and MDSCs induced by GM-CSF or GM-CSF + Dex were added at different ratios between splenic cells and MDSCs for 72 h. The pattern of proliferation was examined by flow cytometric analysis of CFSE dilution in the gated CD4^+^ and CD8^+^ T cells. The percentages indicated CFSE dilution in the gated T cell population (number of splenic cells and MDSCs: 2 × 10^5^ and 0.25 × 10^5^). The inhibitory rates in CD4^+^ T cells **(E)** and CD8^+^ T cells **(F)** were calculated based on the proliferation rates determined by flow cytometry analysis. The data (Mean ± SEM) are representative from three independent experiments (*P < 0.05, **P < 0.01).

To address the role of Dex in the induction of MDSCs, MDSCs were generated from bone marrow cells in the presence of GM-CSF as previously described, with or without different concentrations of Dex for 4 days. As shown in Figure 1A, GM-CSF or GM-CSF with different concentrations of Dex all induced CD11b^+^ Gr-1^+^ cells. Interestingly, we found Dex could increase the percentage and cell number of CD11b^+^Gr-1^int/low^ cells but not CD11b^+^Gr-1^hi^ cells in a dose-dependent manner (Figures 1A,B). Based on the increased percentage and cell number of CD11b^+^Gr-1^int/low^ cells induced by GM-CSF and Dex, CD11b^+^Gr-1^int/low^ cells were gated to further analyze cell surface markers. The expression of CD115, F4/80, and CD11c were identical between the cells treated with GM-CSF or GM-CSF + Dex (Figure 1C), indicating Dex treatment does not alter cell phenotype. Other surface markers indicative for MDSCs suppressive action, including CD80 and CD86 were significantly decreased in cells induced by Dex. CD62L, which enables MDSCs homing into lymphoid organs, was slightly increased on the surface of MDSCs induced by Dex and GM-CSF (Figure 1C).

To directly assess the suppressive function of MDSCs induced by GM-CSF or GM-CSF + Dex, cell proliferation of T cells stimulated by ConA was determined in the presence of the induced MDCs at different ratios. GM-CSF-induced MDSCs can inhibit the proliferation of CD8^+^ and CD4^+^ T cells in a dose-dependent manner, while GM-CSF + Dex-induced MDSCs displayed stronger suppressive capacity (Figure 1D). MDSCs induced by GM-CSF + Dex can inhibit T cell proliferation even in lower ratios (Figures 1E,F). Therefore, these results suggested Dex can enhance the immunosuppressive function of GM-CSF-induced MDSCs *in vitro*.

### The Enhanced Immunosuppressive Function of MDSCs Induced by Dex Is Mediated by iNOS *In Vitro*

**Figure 2:**
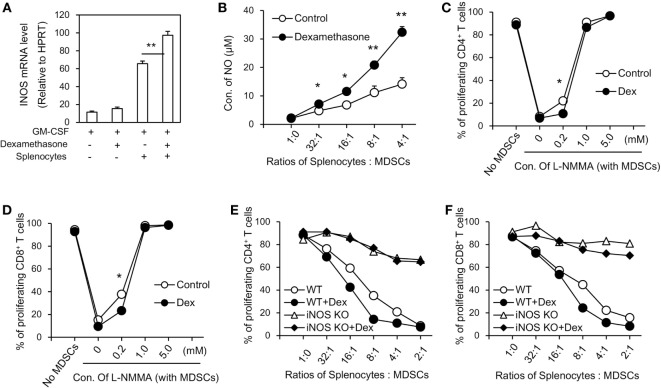
Dexamethasone (Dex) enhances the inhibitory function of myeloid-derived suppressor cells (MDSCs) through iNOS pathway. **(A)** MDSCs (C57BL/6, CD45.1) induced by granulocyte macrophage colony stimulating factor (GM-CSF) or GM-CSF + Dex were cocultured with splenic cells (C57BL/6, CD45.2) in the presence of conA (2 µg/ml) at a ratio of 1:1 for 3 days, and MDSCs were harvested by flow cytometry based on the surface staining of CD45.1. The mRNA expression of *iNOS* in harvested MDSCs was determined by RT-PCR. **(B)** MDSCs induced *in vitro* were cocultured with CFSE-labeled splenic cells (2 × 10^5^ per well) stimulated by ConA (2 µg/ml) at different ratios for 72 h and the NO levels in the coculture medium were assessed. iNOS inhibitor L-NMMA at different concentrations were added into T cells and MDSCs coculture system for 72 h. Percentage of proliferating CD4^+^ T cells **(C)** and CD8^+^ T cells **(D)** were shown. MDSCs induced from iNOS knockout (KO) mice by GM-CSF or GM-CSF + Dex were added to CFSE-labeled splenic cell proliferation assay. iNOS deficiency significantly reversed the inhibitory effect of MDSCs induced by GM-CSF + Dex on CD4^+^ T cells **(E)** and CD8^+^ T cells **(F)**. Data are shown as Mean ± SEM. All the data are composite of three independent experiments (*P < 0.05, **P < 0.01).

To further explore the mechanisms accounting for the enhanced immunosuppressive function seen in GM-CSF + Dex-induced MDSCs, immunosuppressive molecules were assessed in MDSCs induced by GM-CSF or GM-CSF + Dex in the presence or absence of splenocytes. There were no significant differences in potential relevant genes, including Arg1, Nox2, COX2, HO-1, TGF-β, and IDO between MDSCs induced by GM-CSF and by GM-CSF + Dex (data not shown), while the expression of iNOS in GM-CSF + Dex-induced MDSCs was significantly higher than that in GM-CSF-induced MDSCs (Figure 2A). Next, we assessed the level of NO production in MDSCs co-cultured with T cells activated by ConA. The NO production was also significantly higher in the culture system with MDSCs induced by GM-CSF + Dex (Figure 2B), suggesting the critical role of iNOS pathway in the immunosuppressive function of Dex-induced MDSCs. Moreover, addition of a nitric oxide synthase inhibitor, L-NMMA, which can specifically block NO production, significantly reversed the inhibitory effects of MDSCs induced by GM-CSF or GM-CSF + Dex on T cell proliferation in a dose-dependent manner (Figures 2C,D). In addition, there was no significant difference in the immunosuppressive function between iNOS-deficient MDSCs induced by GM-CSF or GM-CSF + Dex, both of which displayed significantly decreased inhibitory function (Figures 2E,F). Inhibition of IDO, Cox2, and Arg1 with their corresponding inhibitors also failed to recover T cell proliferation in the culture system with MDSCs induced by GM-CSF alone or GM-CSF + Dex (data not shown). Taken together, all these results clearly showed that iNOS pathway is responsible for the enhanced immunosuppressive function of MDSCs induced by GM-CSF + Dex in vitro.

### Adoptive Transfer of MDSC-Dex Prolongs Cardiac Allograft Survival in an iNOS-Dependent Manner

**Figure 3:**
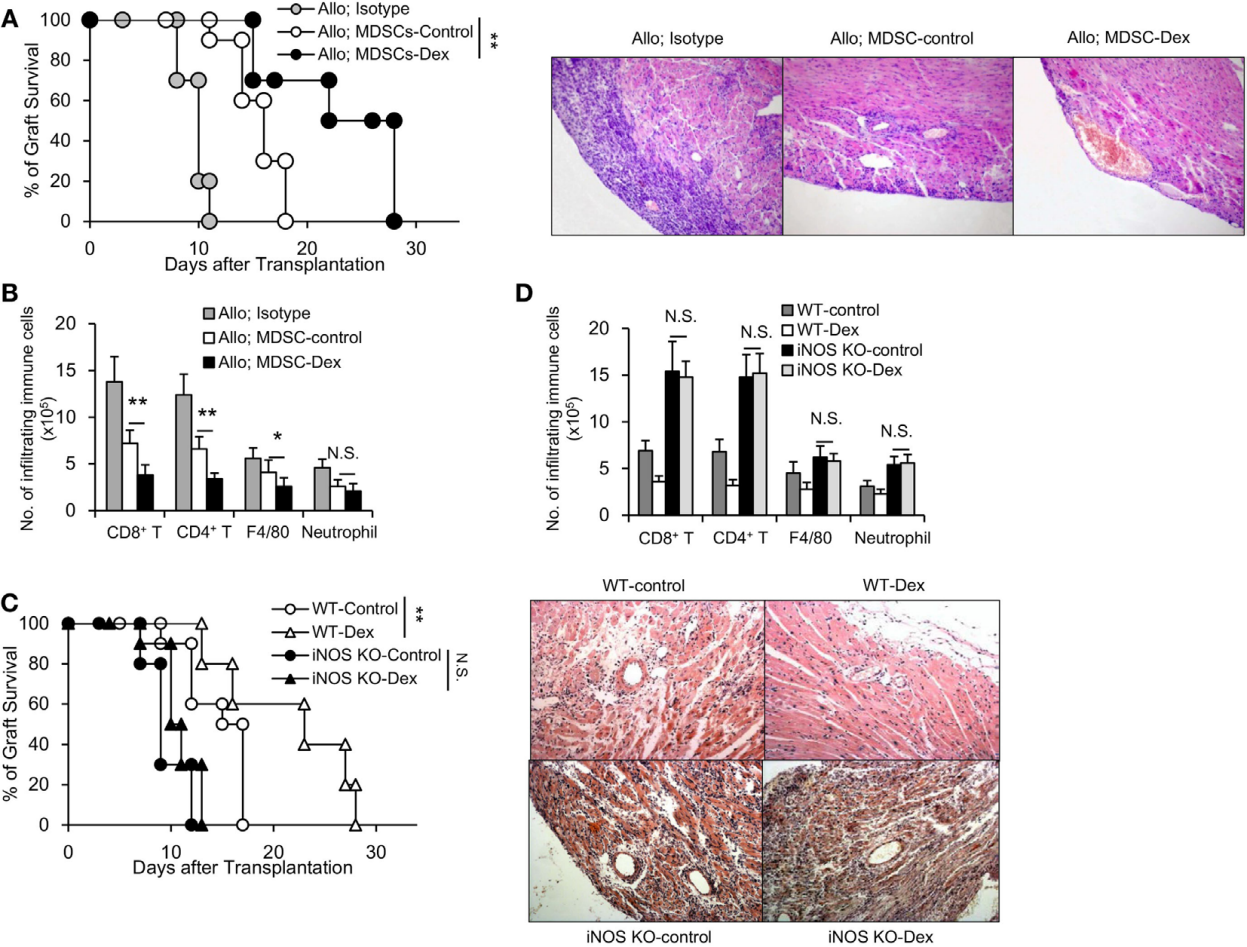
Adoptive transfer of granulocyte macrophage colony stimulating factor (GM-CSF) + dexamethasone (Dex)-induced Myeloid-derived suppressor cells (MDSCs) prolongs cardiac allograft survival in an iNOS-dependent manner. **(A)** Cardiac allograft survival (BALB/c donor hearts to C57BL/6J recipient mice) from mice transferred with sorted CD11b^+^Gr-1^int/low^ MDSCs (3 × 10^6^ per mouse, i.v.) induced by GM-CSF or GM-CSF + Dex immediately after cardiac allograft transplantation was monitored (*n* = 8 per group), and H&E staining of representative sections at postoperative day 7 from mice transferred with MDSCs were shown. **(B)** Numbers of infiltrating immune cells in cardiac allografts were assessed by flow cytometry. **(C)** Deficiency of iNOS reversed the protective function of Dex-induced MDSCs on cardiac allograft in recipient mice (*n* = 8 per group). **(D)** The infiltration of immune cells in cardiac allografts were analyzed in WT or iNOS knockout (KO) mice transferred with MDSCs induced by GM-CSF or GM-CSF + Dex. *P < 0.05, **P < 0.01, N.S., not significant.

To examine the role of MDSCs in allograft rejection *in vivo*, GM-CSF-induced MDSCs (MDSC-control) or GM-CSF + Dex-induced MDSCs (MDSC-Dex) were transferred to allogeneic cardiac transplant mice. Adoptive transfer of MDSC-Dex significantly prolonged cardiac allograft survival when compared to that of MDSC-control (Figures 3A,B). Previous results showed iNOS pathway accounted for the enhanced immunosuppressive function of MDSC-Dex *in vitro*. To explore whether iNOS pathway is also required for MDSC-Dex mediated suppressive function *in vivo*, MDSC isolated from iNOS KO mice were induced by GM-CSF + Dex *in vitro* and were adoptively transferred to allogeneic cardiac transplant mice. Absent of iNOS pathway in MDSC induced by GM-CSF + Dex significantly reversed the effect of MDSC-Dex on allograft survival (Figure 3C). The infiltration of immune cells including CD8^+^ T, CD4^+^ T, and macrophages were also significantly increased in iNOS KO mice transferred with MDSCs induced by GM-CSF + Dex (Figure 3D). Therefore, NO is required for Dex-induced MDSC amelioration of allograft immune response both *in vivo* and *in vitro*.

### MDSCs Induced by Dex Displayed an Enhanced Migration into Allograft through Upregulating the Expression of Glucocorticoid Receptor (GR) and CXCR2

**Figure 4:**
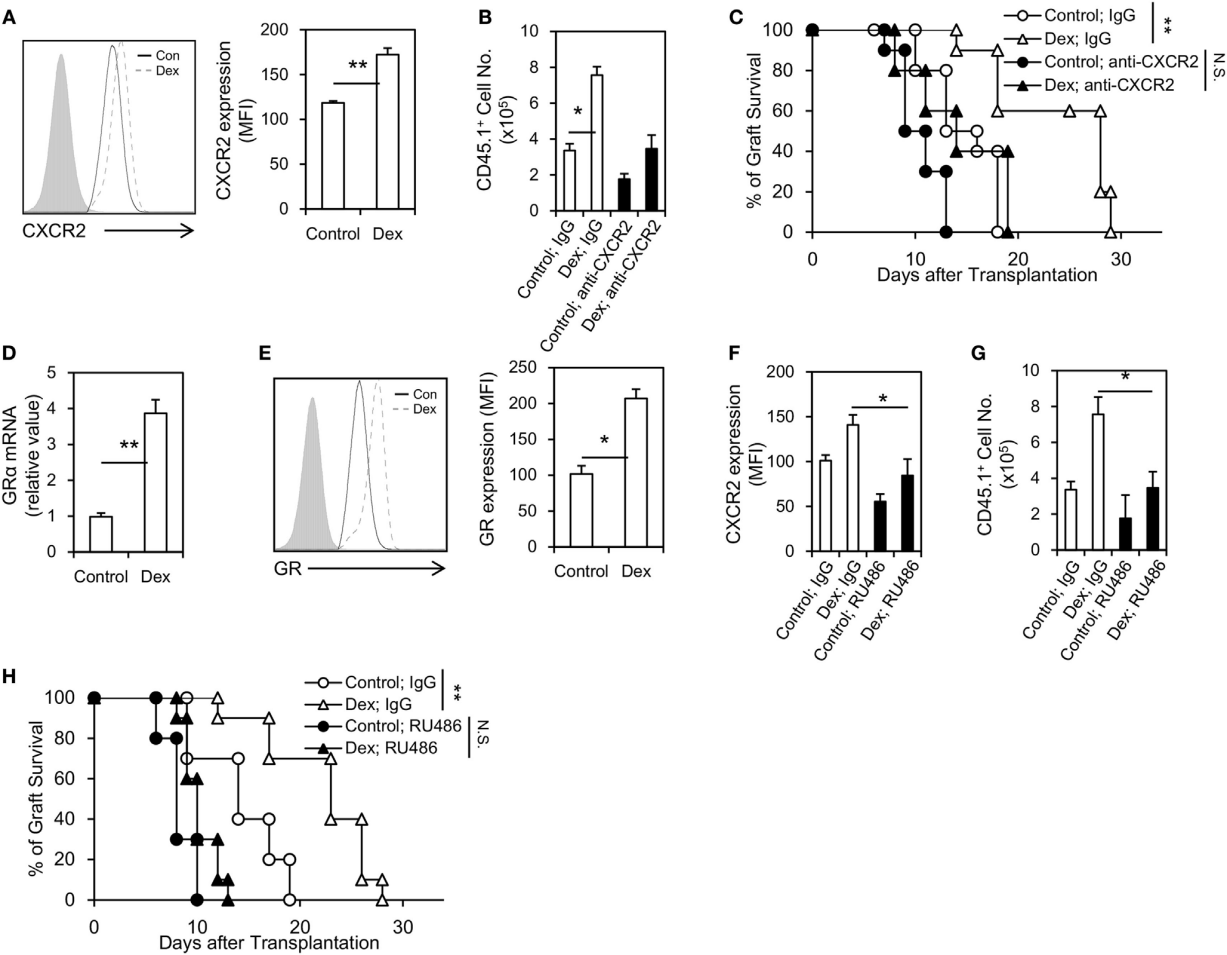
Dexamethasone (Dex) treatment promotes Myeloid-derived suppressor cells (MDSCs) migration into cardiac allograft through upregulating the expression of glucocorticoid receptor (GR) and CXCR2. CXCR2 expression **(A)** in MDSCs induced by granulocyte macrophage colony stimulating factor (GM-CSF) or GM-CSF + Dex was determined by flow cytometry, and the mean fluorescent intensity (MFI) of CXCR2 was summarized. Allograft recipient mice (CD45.2, C57BL6/J) were administered 50 µg neutralizing anti-CXCR2 mAb or IgG isotype control *via* i.v. injection 1 h before transplantation, and then MDSCs induced by GM-CSF or GM-CSF + Dex (3 × 10^6^ per mice, i.v.) from CD45.1 mice (C57BL6/J) were adoptively transferred into these recipients. The infiltrating donor CD45.1^+^ CD11b^+^ Gr-1^+^ cell number **(B)** and graft survival **(C)** were determined (*n* = 8). Dex treatment upregulated the expression of GR in MDSCs both at the protein **(D)** and mRNA levels **(E)**. The GR antagonist RU486 efficiently diminished the CXCR2 expression on MDSCs **(F)** and decreased the infiltration of donor CD45.1^+^ CD11b^+^ Gr-1^+^ MDSCs in cardiac allograft **(G)**. RU486 treatment (10 mg/kg, i.p., 1 h before transplantation) significantly diminished graft survival in recipient received MDSCs induced by GM-CSF + Dex **(H)**. Data (Mean ± SEM) are representative of two independent experiments (*n* = 5–10 in each group). *P < 0.05, **P < 0.01, N.S., not significant.

The migration of MDSCs into allograft is critical for its immunosuppressive function, as well as for the prolongation of allograft survival (18). To explore whether the prolongation of allograft seen in Dex-MDSCs treated mice was due to an enhanced migration of MDSCs, we first assessed the expression of CXCR2, which was implicated in mediating MDSCs recruitment into local inflammatory and tumor sites (19, 20). We found the expression of CXCR2 was significantly upregulated in MDSCs induced by GM-CSF + Dex (Figure 4A), and MDSCs induced by GM-CSF + Dex also displayed a significantly increased infiltration into allograft (Figure 4B). In addition, anti-CXCR2 treatment significantly reduced the number of MDSCs in allograft, resulting in a decreased allograft survival in both MDSCs-Dex group and MDSCs-control group (Figures 4B,C). Thus, Dex promotes the migration of MDSCs into allograft through upregulating the expression of CXCR2.

Next, we explored how Dex modulates the expression of CXCR2 on MDSCs. GR signaling is regarded to be essential for GC effects, and we found Dex treatment significantly upregulated the expression of GR in MDSCs both at the mRNA and protein levels (Figures 4D,E). Moreover, the use of a GR specific inhibitor, RU486, decreased the expression of CXCR2 on MDSCs (Figure 4F). RU486 treatment also reduced the infiltration of MDSCs in allograft (Figure 4G), leading to a diminished allograft survival (Figure 4H). Taken together, these results suggest Dex promotes the migration of MDSCs into allograft through GR-dependent pathway.

### Dex-Induced MDSCs Promote CD4^+^ Foxp3^+^ Treg Expansion

**Figure 5:**
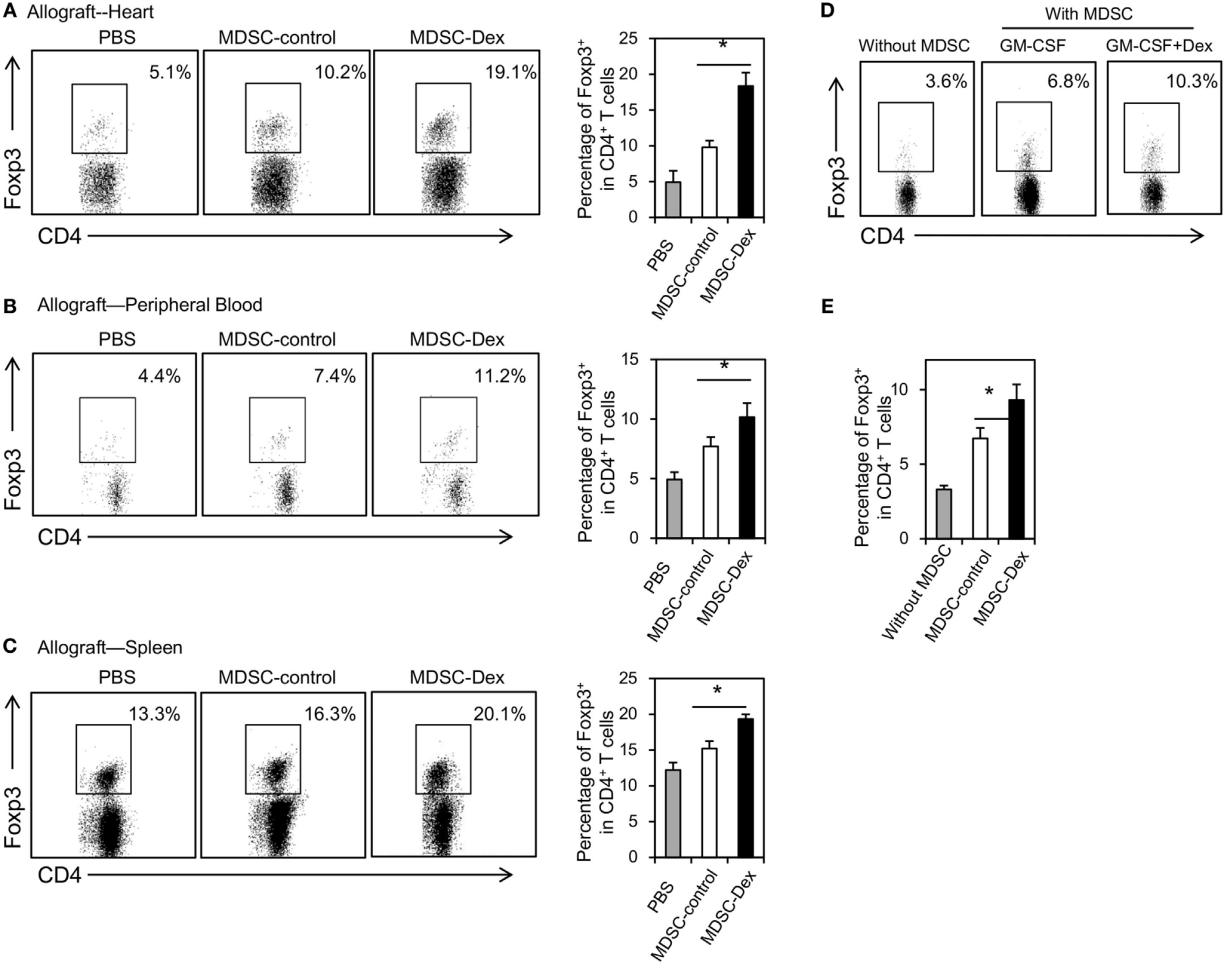
Adoptive transfer of myeloid-derived suppressor cells (MDSCs) induced by granulocyte macrophage colony stimulating factor (GM-CSF) and dexamethasone (Dex) favors the expansion of CD4^+^ Foxp3^+^ regulatory T cells (Treg) *in vivo*. MDSCs induced by GM-CSF or GM-CSF + Dex (3 × 10^6^ per mice) were adoptively transferred into recipient mice *via* the tail vein immediately after cardiac allograft transplantation. On post transplantation day 7, recipient mice were euthanized and the percentages of CD4^+^ Foxp3^+^ Treg in allograft **(A)**, peripheral blood **(B)**, and spleen **(C)** were assessed. **(D,E)** MDSCs (CD45.2, C57BL6/J) induced by GM-CSF or GM-CSF + Dex mice were cocultured with CD4^+^ T cells (CD45.2, C57BL6/J) purified by flow cytometry from the spleen at a ratio of 1:4 in the presence of anti-CD3 (5 μg/ml)/CD28(2 µg/ml) for 3 days. The percentages of Foxp3^+^ cells in CD4^+^ T cells were assessed. Data are Mean ± SEM. All the data are composite of three independent experiments (*n* = 3–5 in each group). *P* values were determined by Student’s *t*-test. *P < 0.05, N.S., not significant.

Previously, MDSCs have been shown to mediate CD4^+^ Foxp3^+^ Treg development in mouse tumor models *in vivo* (21, 22). Recent studies further suggested that MDSCs can induce Treg development in animal transplantation model (23) and renal transplant patients (24). To address whether MDSCs in our model can also promote the expansion of Treg *in vivo*, the percentages of Treg in the allografts, the peripheral blood and the spleens of mice receiving MDSC-control or MDSC-Dex were analyzed at post transplantation day 7. As shown in Figure 5A, the percentage of Treg in the allografts of mice receiving MDSC-Dex was significantly higher than that receiving MDSC-control. Similar results were also found in the peripheral blood and the spleen (Figures 5B,C). To direct assess the effect of MDSCs on the expansion of Treg, we cocultured MDSCs induced by GM-CSF or GM-CSF + Dex with purified CD4^+^ T cells from the spleen for 3 days. As shown in Figures 5D,E, MDSCs induced by Dex + GM-CSF significantly enhanced the pool of Treg. Taken together, all these results suggested MDSCs induced by GM-CSF + Dex favors the expansion of Treg.

## Discussion

Historically, developing therapeutic strategies of transplantation tolerance mainly focuses on targeting adaptive immune response, based on the findings that T cells are both necessary and sufficient to induce allograft rejection (25–28). Deletion of activated T cells and development of Treg are the two main mechanisms. However, deletion of activated T cells is necessary but not sufficient for the induction of immune tolerance and Treg also do not seem to contribute to immune tolerance during the early phase (29, 30). Modulation of innate immune cells may be a candidate to resolve this problem. Previous studies identified that classic antigen-presenting cell dendritic cells can serve as a potent innate immune cell population which promotes transplantation tolerance through inducing antigen-specific Treg (31–35). Recently, accumulating evidence from animal models also suggest a great potential of MDSCs for preventing allograft rejection (6, 36, 37), which may be a new potential therapy target on innate immune cells to control graft rejection in the clinic.

To develop a clinically acceptable method through manipulating MDSCs, *in vitro* induction of potent and stable MDSCs has been tried (6, 7). Dex is a classic drug used in clinical trials to ameliorate immune-mediated allograft rejection associated with inflammation, and can modulate immune cell phenotype and function (11–13). Recently, systemic administration of Dex has been shown to modulate MDSCs expansion *in vivo* both in skin transplantation (16) and renal disease model (38). Dex can also favor the migration of MDSCs into inflammatory sites to control immune response (16). In addition, Dex modulates MDSCs immunosuppressive function through GR signaling in an immunological hepatic injury model (39). Therefore, Dex is an important regulator of MDSCs expansion and/or function. However, since Dex can also modulate GR signaling in other cells (40, 41), systemic injection of Dex may not be an ideal way to manipulate MDSCs in future clinical trials. In our study, we used Dex combined with classic MDSC induction cytokine GM-CSF to generate the differentiation of MDSCs *in vitro*. We found Dex can specifically increase the cell number of CD11b^+^ Gr-1^int/low^ MDSCs *in vitro* with more powerful immune suppressive function on T cell proliferation. Mechanistic study showed that iNOS pathway is required for the immune regulatory function of Dex-induced MDSCs as significantly increased production of NO was found in Dex-induced MDSCs system. In addition, inhibition of NO production with genetic deletion of iNOS or chemical inhibitor blocked the immunosuppressive function of Dex-induced MDSCs *in vitro*. Consistent with these *in vitro* results, adoptive transfer of Dex-induced MDSCs significantly prolongs the survival of cardiac allograft *in vivo*.

Glucocorticoid receptor is one of the key mechanisms for the effect of Dex on MDSCs. Modulation of GR by Dex can suppress HIF1α and HIF1α-dependent glycolysis in MDSCs which promotes the immune suppressive activity of MDSCs (39). On the other hand, pharmacological inhibition of GR by RU486 can also suppress the function of MDSCs, leading to the diminished allo skin graft survival (16). To explore whether GR signaling is also involved in our study, we first assessed the GR level in MDSCs. We found GR expression was significantly increased in Dex-induced MDSCs, indicating the importance of GR signaling in Dex-induced MDSCs. The migration of MDSCs into inflammation site, i.e., the allograft in transplantation model, mainly depends on surface expression of CXCR2, which also plays a critical for immune tolerance. We found Dex treatment increased the expression of CXCR2 on MDSCs. Importantly, blockade of CXCR2 with a CXCR2 mAb or blockade of GR with its specific inhibitor RU486 significantly decreased the expression of CXCR2 on MDSCs and also recruitment of MDSCs into allograft, leading to a diminished survival of allograft survival. Taken together, these results demonstrated that GR signaling is required for the immune regulation of Dex-induced MDSCs.

Myeloid-derived suppressor cells and Treg are both important immune regulator cells in the context of organ transplantation (42). Since Treg or MDSCs alone was not sufficient to induce long-term tolerance, it was attractive to investigate interaction between MDSCs and Treg. Indeed, several studies have shown MDSCs promote the development and homeostasis of Treg in tumor (21, 22, 43) and organ transplantation (1, 18). Moreover, the interaction of Dex, MDSCs, and Treg has been identified in a focal segmental glomerulosclerosis mice mode (38). Both systemic administration of Dex or adoptive transfer of MDSCs can increase the percentages of Treg in the spleen and the kidney, and pharmacological depletion of MDSCs resulted in decreased levels of Treg (38). However, this study did not assess the direct effect of MDSCs on Treg expansion *in vitro*, and the renal injury itself can trigger strong expansion of Treg expansion (38). Thus, it is possible that depletion of MDSCs may lead to the changes of other cells which have an indirect impact on Treg expansion. We also investigated whether adoptive transfer of Dex-induced MDSCs can have an impact on Tregs *in vivo*. Consistent with other studies, adoptive transfer of Dex-induced MDSCs can favor the expansion of Treg both systemically and in allograft *in vivo*. Detailed mechanisms for the expansion of Treg upon adoptive transfer of Dex-induced MDSCs are not clear. Several mechanisms have been suggested to promote the expansion of Treg by MDSCs, including chemokine driven attraction of Treg (43, 44), and induced proliferation of natural Treg (45, 46). We identified a systemic expansion of Treg after adoptive transfer of MDSCs with an increased number of total Treg pool (data not shown), and *in vitro* culture of MDSCs with CD4^+^ T cells can promote the expansion of Treg. Therefore, in the context of our model, MDSCs may directly expand the population of Treg through one or combined mechanisms mentioned above. Molecules responsible for the expansion of Treg by MDSCs includes iNOS signaling (1), CD274 (46), arginase (45), and IL-10 (22). In our study, no significant differences in CD274, Arg1 and TGF-β levels were found between MDSCs induced by GM-CSF or GM-CSF + Dex (Figure 1C, data not shown). Blockade of iNOS signaling in MDSCs with the use of iNOS KO mice also did not have an impact on the expansion of CD4^+^ Foxp3^+^ T cells *in vivo* (data not shown). The production of IL-10, which was shown to maintain Foxp3 expression in Treg (47) and also potentiated differentiation of human induced Treg (48), was significantly increased in Dex-induced MDSCs (data not shown). Future studies on the molecules responsible for the expansion of Treg are urged to fully address these questions.

In summary, we have shown that Dex treatment can enhance the expansion and immunosuppressive function of *in vitro*-generated MDSCs. Adoptive transfer of these Dex-induced MDSCs *in vitro* can also prolong cardiac allograft survival and favor the expansion of Treg *in vivo*, which may be a new approach of cellular therapy for organ transplantation.

## Ethics Statement

All the animal experiments were carried out following the Guidelines for the Care and Use of Laboratory Animals of Nanjing University, and were approved by the ethical review committee of Nanjing University.

## Author Contributions

J-FD and Y-tD conceived and designed the experiments; X-FS, YZ, and KC performed the experiments; JD did the allogenic cardiac transplantation; X-FS and XK analyzed the data; J-FD and W-xG contributed reagents/materials/analysis tools; B-rL designed the experiments of Treg induction *in vitro*, X-FS wrote the paper.

## Conflict of Interest Statement

The authors declare that the research was conducted in the absence of any commercial or financial relationships that could be construed as a potential conflict of interest.
